# Scenario-based simulation training for the WHO hand hygiene self-assessment framework

**DOI:** 10.1186/s13756-019-0511-9

**Published:** 2019-03-28

**Authors:** Ermira Tartari, Carolina Fankhauser, Alexandra Peters, Buyiswa Lizzie Sithole, Funda Timurkaynak, Sarah Masson-Roy, Benedetta Allegranzi, Daniela Pires, Didier Pittet

**Affiliations:** 1 Infection Control Programme and WHO Collaborating Centre on Patient Safety, University of Geneva Hospitals and Faculty of Medicine, 4 Rue Gabrielle-Perret-Gentil, 1211 Geneva 14, Switzerland; 2 Institute of Global Health, Faculty of Medicine, University of Geneva, Geneva, Switzerland; 3 Department of Nursing, Faculty of Health Sciences, University of Malta, Msida, Malta; 4 Infection Control Africa Network, Unit of IPC, Tygerberg Hospital, Cape Town, South Africa; 5 Infection Prevention and Control Global Unit, Department of Service Delivery and Safety, World Health Organization, Geneva, Switzerland; 6 Department of Infectious Diseases, Centro Hospitalar Lisboa Norte and Faculdade de Medicina da Universidade de Lisboa, Lisbon, Portugal

**Keywords:** Simulation, Education, Infection prevention and control, Hand hygiene, Healthcare-associated infection, Antimicrobial resistance, Multimodal promotion, Universal health coverage, Quality, Patient safety, World Health Organization

## Abstract

The WHO *SAVE LIVES: Clean Your Hands* global hand hygiene campaign, launched in 2009 and celebrated annually on the 5th of May, features specific calls to action seeking to increase engagement from stakeholders’ collaborations in hand hygiene improvement. WHO calls on everyone to be inspired by the global movement towards universal health coverage (UHC). Infection prevention and control (IPC), including hand hygiene, is critical to achieve UHC as it has a direct impact on quality of care and patient safety across all levels of the health services. In the framework of UHC, the theme for 5 May 2019 is “Clean care for all – it’s in your hands”. In this context, the WHO has launched a global survey to assess the current level of progress of IPC programmes and hand hygiene activities in healthcare facilities (HCFs) worldwide. This involved the creation of two tools for healthcare facilities: the WHO Infection Prevention and Control Assessment Framework (IPCAF) and the WHO Hand Hygiene Self-Assessment Framework (HHSAF). The objective of this paper is to provide case scenario-based simulation for IPC specialists to simulate and fully assimilate the correct completion of the HHSAF framework in a standardized format. The three case scenarios have been tested and are proposed for the reader to assess the HHSAF of different HCFs in a variety of contexts, even in low-resouce settings. They were designed for simulation training purposes to achieve standardization and interactive learning. These scenarios are meant to be used by professionals in charge of implementing a hand hygiene improvement strategy within their HCF, as well as for simulation and standardized training purposes prior to completing and submitting data for the 2019 WHO Global Survey. Additionally, information provided by the use of the HHSAF can easily be translated into action plans to support the implementation and improvement related to specific indicators of hand hygiene promotion. We invite all HCFs to participate in the 2019 WHO global survey and monitor the level of progress of their IPC programme and hand hygiene activities.

## Background

Too many of the vulnerable individuals admitted to health-care settings develop a health-care associated infection (HAI). This results in increased morbidity and mortality, prolonged hospital stay, and financial losses for health care systems [1, 2]. Additionally, prevention of transmission and control of multidrug-resistant organisms in health care settings are critical as the number of antibiotics available to treat these infections is limited [3]. Many of these issues could be prevented through simple, low-cost infection prevention and control (IPC) measures such as hand hygiene performed at critical moments [4, 5].

The focus of the World Health Organization (WHO) *SAVE LIVES: Clean Your Hands* global campaign has been to promote best hand hygiene practices as a key component of achieving quality of care and patient safety [6–11]. This campaign, launched in 2009 and celebrated annually on the 5th of May, features specific calls to action each year, and seeks to increase engagement from stakeholders’ collaborations in strenghtening IPC programmes and improving hand hygiene. This year’s campaign theme is “Clean care for all – it’s in your hands” [11] (https://www.who.int/infection-prevention/campaigns/clean-hands/en/).

‘Health for All’ has been increasingly recognized in international fora as a concept firmly based on equity. Alongside a strong global momentum surrounding universal health coverage (UHC), WHO calls on everyone to contribute to the attainment of health for all populations [12]. In the context of UHC, IPC with hand hygiene as a fundamental measure, is a key component in providing patient safety and high quality health services [12, 13].

### The 2019 WHO global survey

The WHO global survey aims to assess the current level of progress of IPC programmes and hand hygiene activities in HCFs in the context of the WHO *SAVE LIVES: Clean Your Hands* annual hand hygiene global campaign (https://www.who.int/infection-prevention/campaigns/ipc-global-survey-2019/en/). The survey will be open until July 2019, and WHO invites all HCFs to join. The survey is based on the use of two tools at the HCF level: the WHO Infection Prevention and Control Assessment Framework (IPCAF) (https://www.who.int/infection-prevention/tools/core-components/IPCAF-facility.PDF?ua=1) and the WHO HHSAF (https://www.who.int/gpsc/country_work/hhsa_framework_October_2010.pdf?ua=1) [14, 15]

The IPCAF is a validated assessment tool that supports the implementation of the WHO recommendations on the core components of effective IPC programmes [16] at the acute HCF level. The goal of the framework is to assess the current IPC situation in HCFs. It is especially focused on evaluating existing IPC activities/resources, and identifying strengths and gaps that can inform future policies. It can be considered as a diagnostic tool for HCFs to detect relevant problems or shortcomings that require improvement as well as identify areas where international standards and requirements can be met [16, 17].

The HHSAF is a systematic tool with which an individual health-care facility can obtain a situational analysis of its hand hygiene promotion and practices [14, 15]. To guide future improvement WHO is launching the HHSAF again in 2019 as part of the WHO global survey (the HHSAF was previously launched in 2011 and 2015) (https://www.who.int/infection-prevention/campaigns/ipc-global-survey-2019/en/).

### Assesing the level of hand hygiene at your institution

Measuring, promoting, improving, and subsequently sustaining hand hygiene standards as quality indicators for patient safety is essential [18]. To advance this agenda, it is crucial to monitor where improvements have occurred and gaps must be addressed in hand hygiene structures, processes, resources, promotion and practices [19, 20].

WHO provides a range of tools and resources to sustain hand hygiene improvement. One is the Hand Hygiene Self-Assessment Framework (HHSAF), which is a validated tool used to quantify the status of hand hygiene promotion activities within healthcare facilities worldwide [14, 15]. Launched by WHO in 2010, the HHSAF is available in different languages; it remains the most widely used tool and the only framework aimed at tracking the level of progress of healthcare facilties in the context of hand hygiene implementation. Structured around the five components of the WHO Multimodal Hand Hygiene Improvement Strategy (see Appendix), the HHSAF assesses the interventions implemented by HCFs in the context of their adherence to WHO hand hygiene guidelines and recommendations [18–20]. The HHSAF analyses a number of factors within each of the five components of the multimodal strategy and scores institutions’ status as inadequate, basic, intermediate and advanced, according to the number of points obtained (see Appendix). Additionally, it directs HCFs to the WHO hand hygiene promotion tools and the template action plans (available at https://www.who.int/infection-prevention/tools/hand-hygiene/en/) that can be used to make improvement plans according to the HHSAF score and specific indicators identified as requiring attention.

Given this important focus on hand hygiene in IPC programmes, the WHO previously conducted two global surveys using the HHSAF in 2011 and 2015, inviting HCFs worldwide to submit their self-assessments [21]. The two surveys offered a bird’s eye view of hand hygiene programmes at the HCF level and enabled better comparisons across regions and over time, prompting calls for further improvements. In 2011, the majority of HCFs participating were from developed countries and reflected an intermediate level of progress. When the survey was repeated in 2015, the overall score increased significantly (*p* < 0.001) in the 86 HCFs that participated in both surveys [21]. Improvement was documented in all regions, particularly in the Eastern Mediterranean region, Europe and the Americas. The African region scored lowest, which could be indicative of a poorer IPC infrastructure, resources and basic knowledge in hand hygiene implementation and issues concerning sustainability. The disparities that emerged from the two global HHSAF surveys emphasize the need for additional improvement of hand hygiene practices, especially in low-resource settings.

### Supporting hand hygiene education and training

To support education and training activities around the global survey, WHO, together with the WHO Collaborating Centre on Patient Safety, developed a body of material including an educational video (available at https://www.youtube.com/watch?v=PDz8kxrPaMk&feature=youtu.be), a promotional video (available at https://www.youtube.com/watch?v=UfH6ODLV3BI) and three case scenario-based simulations (Tables 1, 2 and 3) for IPC specialists to simulate and fully assimilate the correct completion of the HHSAF framework in a standardized format. Simulation in health care is widely used in medical education as an active learning method and it has been shown to have great potential [22–24].

**Table 1:** Case Scenario 1 to simulate the completion of the WHO Hand Hygiene Self Assessment Framework (HHSAF)

Case Scenario	HHSAF Component	Subtotal Score
The Bellevue University Medical Centre is a tertiary care institution with 1000 beds and three separate campuses.		
Hand hygiene (HH) products including alcohol-based hand rubs (ABHR) are available facility-wide with continuous supply and at the point of care. There are one to three sinks in every patient’s room together with non-medicated soap, paper towels and alcohol-based hand rub dispensers with proven efficacy and tolerability.	System Change	90/100
The HH promotion strategy is based on the World Health Organization (WHO) 5 Moments for Hand Hygiene and includes mandatory HH training upon employment and at least annually for all health workers by trained and validated Infection Prevention and Control (IPC) practitioners. All WHO training materials are made available in the hospital’s intranet. Non-attendance is directly linked to closed access to the hospitals’ informatics systems.	Training and Education	90/100
Availability of HH products (ABHR, soap, single use paper towels) is audited on a regular basis. A quarterly schedule of HH compliance monitoring has been established (Periods 1–4), and is conducted by validated IPC practitioners. Immediate feedback to health workers is encouraged. In 2012, 10,000 HH opportunities and 3740 actions were observed. In 2017, 10,000 HH opportunities and 6700 actions were observed. HH compliance before patient contact is 10–15% lower than after patient contact. HH compliance was highest among nursing staff (73.6%) and lower among medical staff (52.3%). The use of ABHR accounts for the majority of HH actions performed in the facility.	Evaluation and Feedback	75/100
Visual reminders in the form of posters on “My 5 Moments for Hand Hygiene” and HH technique are displayed in strategic clinical areas within the health care facility. On admission, patients are provided with a brochure about the importance of HH and posters promoting patient participation are displayed in patient areas. There is no system in place to update posters regularly, however.	Reminders in the workplace	47.5/100
The IPC/hand hygiene team (one full-time doctor and five full time nurses) have been implementing a HH culture-change program for the past five years, spearheaded by hospital’s leadership and leading a country wide national HH initiative aiming to improve health care workers’ HH compliance, increase use of ABHR and reduce HAIs. The hospital celebrates the world HH day on the 5th of May. A process that provides HH compliance performance feedback (every six months) is in place, and is driven and supported by the hospital leadership. High performing wards are publicly recognized within the hospital and their HH compliance levels set the HH targets for the following year. The hospital has a system of HH champions in all medical, surgical and high-risk wards.	Institutional Safety Climate	65/100
Bloodstream infections (BSI), surgical site infections (SSI) and MRSA clinical cultures are monitored in high-risk areas and facility wide, and a point prevalence survey of HAIs is performed annually. A decrease in overall HAIs (prevalence of 17.3% in 2000 to 9.4% in 2015) was reported, MRSA transmission rates decreased (2.16 to 0.93 episodes per 10,000 patient-days), and the consumption of ABHR increased from 12.5 to 22.4 L per 1000 patient-days in the past five years. HAI data are presented regularly to hospital leadership and to health workers together with HH compliance rates.	Leadership	10/20
	Total Score	377.5/500

*Abbreviations*: *ABHR* Alcohol-based handrub, *BSI* Bloodstream infections, *HAIs* Healthcare-associated infections, *HH* Hand hygiene, *MRSA* Methicillin-resistant *Staphylococcus aureus, SSI* Surgical site infections

**Table 2:** Case Scenario 2 to simulate the completion of the WHO Hand Hygiene Self Assessment Framework (HHSAF)

Case Scenario	HHSAF Component	Subtotal Score
St. Mary’s Teaching Hospital is a large tertiary care institution with 550 beds in rural Uganda. The hospital has 24 clinical wards, including radiology, laboratory, and pharmacy services.		
One-liter ABHR bottles are mounted on the walls of the wards for ease of access. Mobile bottles are also placed on the trolleys used for ward rounds, in the reception area and in the treatment room area. The ABHR used was locally produced from sugar cane. Local ABHR production and quality control have proven to be feasible and satisfactory. Hand hygiene supplies are scarce. Only one or no functional sinks/taps were available in each ward. Portable water bottles and basins are an alternative for handwashing. Gloves, even non-sterile ones, are rarely available. An action plan has recently been developed to improve the infrastructure in the hospital.	System Change	10/100
HH education and training for health workers is sporadic and mainly occurs upon initial employment. The training is provided by the IPC nurse who has received training from the Infection Control Africa Network (ICAN)	Training and Education	20/100
HH compliance rate is monitored by direct observations using the WHO hand hygiene monitoring tool, however there is no established HH promotion strategy; observations are conducted annually. The HH compliance rate was very low. In 2015, the overall reported compliance was 9.2%, but by 2017 it had increased to 21.8%.	Evaluation and Feedback	10/100
The WHO ‘How to handwash’, ‘How to handrub’ and ‘My 5 Moments for Hand Hygiene’ posters were only available in some of the hospital wards.	Reminders in the workplace	25/100
No IPC team, or experts in infection control is available in the country, although one part-time infection control nurse has been assigned infection control duties in the hospital. The director of nursing is committed to supporting hand hygiene improvement at St Mary’s hospital. Since 2017, the hospital is engaged in the WHO *Save Lives Clean Your Hands* annual campaign and is celebrating the 5th of May global HH day.	Institutional Safety Climate	15/100
	Total Score	80/500

*Abbreviations*: *ABHR* Alcohol-based handrub, *HH* Hand hygiene

**Table 3:** Case Scenario 3 to simulate the completion of the WHO Hand Hygiene Self Assessment Framework (HHSAF)

Case Scenario	HHSAF Component	Subtotal Score
The Ulwazi National Referral Hospital is a national referral and teaching tertiary care institution with 900 beds providing both primary and specialized health care (maternity, hemodialysis, intensive care units, medical and surgical specialties).		
HH supplies (i.e. ABHR, soap and disposable towels) are scarce in the facility, with the availability of ABHR being confined to specialty areas such as Maternity, Hemodialysis and Intensive Care Units. The ABHR efficacy and tolerability have not been proven. The other wards use soap for handwashing and do not have disposable paper towels. Soap supplies are erratic and, at times, not available. The soap is very harsh to the skin. The wards have sinks with running water. The sinks are installed in the following areas in the ward: • 1 in the nursing station• 1 in the procedure/treatment room• 2 sinks per ward (25 beds) • 1 sink for the patient wash and toilet areas	System Change	10/100
HH training is done as part of in-service education, upon employment. When newly employed staff members are trained in HH, they enter their names and signatures in a register, kept as a record that all HCWs completed the training. Student nurses are given the HH training in their 1st and 3rd year of undergraduate education. The student nurses are attached for clinical practice in the hospital and have contact with the patients from the first year. The WHO leaflets on ‘Glove Use Information’ and “Hand Hygiene: Why, How and When” are available.	Training and Education	50/100
Audits to assess the availability and consumption of HH products is not performed, as the hospital lacks human resources. HH compliance rates are not monitored. The IPC nurse has not been trained as a hand hygiene compliance observer.	Evaluation and Feedback	0/100
Posters displaying the WHO ‘My 5 Moments of Hand Hygiene’, ‘How to Hand Rub’ and ‘How to handwash’ are displayed next to all the hospital’s sinks.	Reminders in the workplace	50/100
The Ulwazu facility has registered for the WHO *Clean Care is Safer Care* global campaign and is commemorating the Hand Hygiene Day every 5th of May.	Institutional Safety Climate	10/100
	Total Score	120/500

*Abbreviations*: *ABHR* Alcohol-based handrub, *HH* Hand hygiene

The case scenarios presented here can be used by professionals in charge of implementing a hand hygiene improvement strategy within their facility, as well as for simulation and standardized training purposes prior to completing and submitting data for the 2009 WHO Global Survey. As proposed by the WHO Guide to Implementation, information provided through the use of the HHSAF should be translated into action plans to support the implementation and improvement related to specific indicators of hand hygiene promotion [19, 20]. HCFs worldwide should consider implementing a system of continuous assessment of hand hygiene improvement strategy [21] by utilizing the HHSAF tool systematically; the current recommendation by WHO and experts is to use it at least annually.

Information appropriate for each of the WHO Multimodal Hand Hygiene Improvement Strategy components has been identified in the case scenarios (Tables 1, 2 and 3). In order to simulate the completion of the HHSAF, they provide details regarding HH promotion strategy for the evaluation of resources, promotion, and practices within a given HCF. The three case scenarios took in consideration a range of HCFs around the world in a variety of contexts, regardless of the resources available. Drawing from the information provided, a score is assigned for each component and section. The sum of the maximum values in each section is 100 points, adding up to a maximum overall score of 500 points. Based on the overall score, a HCF is then assigned to one of four levels of hand hygiene implementation progress (see Appendix). The HCF presented in Table 1 scored as *Advanced*, and can therefore undergo further *Leadership* assessment according to twenty additional criteria, but since only ten of them have been met, the HCF is not classified as having reached the *Leadership* level. Information for baseline evaluation of hand hygiene activities within a lower income setting is presented in Table 2; based on the overall score, the HCF would be assigned as *Inadequate* level of progress. The HCF in Table 3 is assigned to *Basic* level, identifying the need for significant improvement. The use of HHSAF over time will enable HCFs to keep track of their progress and continuously set new targets for improvement.

## Conclusion

The foremost objective of the WHO global annual campaign is to prioritize IPC in health care and the global health agenda, with hand hygiene assessment and improvement playing a fundamental role in ensuring patient safety. We invite all HCFs to be actively engaged in this important endeavour and call upon all those who can contribute towards achieving high-quality health care and universal health coverage for the improvement of public health worldwide.

“Clean care for all – it’s in your hands”

## Appendix

### Hand Hygiene Self-Assessment Framework

**Table 4:** Levels of Hand Hygiene Implementation Progress Defined by the WHO Hand Hygiene Self-Assessment Framework (HHSAF)

Level of Progress	HHSAFScore	Definition
Inadequate	0–125	Hand hygiene practices and promotion are deficient. Significant improvement is required
Basic	126–250	Some measures are in place, but not at a satisfactory standard. Further improvement is required
Intermediate	251–375	An appropriate hand hygiene promotion strategy is in place, and hand hygiene practices have improved. It is now crucial to develop long-term plans to ensure that improvement is sustained and progresses
Advanced	376–500	Hand hygiene promotion and optimal hand hygiene practices have been sustained and/or improved, helping to embed a culture of safety in the healthcare setting
Leadership^a^		The healthcare facility is considered as a reference center for and contributes to the promotion of hand hygiene through research, innovation and information sharing

^a^The healthcare facility reached the Advanced level and in addition meets at least 12 out of 20 leadership criteria and at least one leadership criterion per each category

## Abbreviations

HAI: Health-care associated infection
HCF: Health care facility
HHSAF: Hand Hygiene Self-Assessment Framework
IPC: Infection Prevention and Control
IPCAF: Infection Prevention and Control Assessment Framework
UHC: Universal Health Coverage
WHO: World Health Organization
